# Mechanics of Corrugated and Composite Materials

**DOI:** 10.3390/ma15051837

**Published:** 2022-03-01

**Authors:** Tomasz Garbowski

**Affiliations:** Department of Biosystems Engineering, Faculty of Environmental and Mechanical Engineering, Poznan University of Life Sciences, Wojska Polskiego 50, 60-627 Poznan, Poland; tomasz.garbowski@up.poznan.pl

The main aim of this Special Issue in Materials was to collect interesting and innovative works on the mechanics of corrugated and composite materials. Corrugated core materials are increasingly used as structural materials or load-bearing elements in a variety of lightweight engineering structures. Due to the specific composition of the composite layers of corrugated materials, the ratio of their load capacity to the weight of sections is much higher than in the case of traditional solid sections. In addition, the geometries of corrugated structures proposed by scientists from around the world are constantly modified to improve their mechanical properties. Composite materials, due to their unique design properties, can be used in many areas to solve difficult problems where traditional materials often fail.

In this Special Issue, the most interesting research papers on various aspects of this broad research field have been collected. From theoretical issues related to the influence of transversal shear on the parameters of corrugated cardboard, to experimental and numerical analysis of an aluminum structure protecting against the effects of an explosion. By enabling scientists and engineers to present the latest knowledge on advances in theoretical, experimental and computational approaches for corrugated and composite materials, it was possible to present a very comprehensive set of research papers.

In research work [1], the authors were focused on the numerical homogenization of plates with a periodic core. The periodicity of the soft core in this case was related to the sinusoidal shape of the middle layer of the multilayer structure made of cardboard. In these types of plates, the transversal shear has a very large influence on their mechanics. A traditional assumption based on the Kirchhoff–Love theory fails and the Reissner–Mindlin theory must be used. The authors presented an extension of the existing homogenization method based on the elastic equilibrium of the strain energy by including the effects related to transversal shear. This method uses the principles of finite element modeling; however, it does not require any formal numerical analysis. The heart of this approach is the matrix linking the effective strains with displacements in the outer nodes of the representative volumetric element (RVE), and the stiffness matrix of the entire RVE condensed to these nodes.

In article [2], the authors were focused on the mechanics of corrugated cardboard. The aim of the work was to derive simplified predictive models to identify the total stiffness and compressive strength of corrugated board samples. The authors used a non-contact method of measuring deformation on the sample surface, based on virtual optical strain gauges, thus eliminating the unreliable measurement of displacement in the standard edge crush test. Video extensometry was used to collect measurements from the outer surfaces of the sample on both sides. As a representative example in this study, an unsymmetrical five-layer sample with two corrugated layers was used. Reliable determination of the stiffness of multilayer structures made of thin panels is not an easy task because buckling of the panels quickly occurs in this type of section and must be taken into account in the calculations. The authors proposed a very effective analytical model for determining the compressive strength of corrugated board based on video extensometric measurements and taking into account preliminary buckling.

The edge compression response was also analyzed in paper [3], which investigated a composite structural insulating panel (CSIP) with magnesium oxide plate facings. The authors studied a novel multifunctional sandwich panel introduced into residential construction as part of wall, floor and roof assemblies. The study was conducted to build a computational tool for the reliable prediction of CSIP failure modes subjected to various axial loads, both concentric and eccentric. The paper proposed an advanced numerical model (based on the finite element method), which takes into account geometric and material nonlinearities, and also takes into account the effect of bimodularity of the material. Additionally, the model was verified by means of laboratory tests on small-scale CSIP samples with three different slenderness ratios and full-size panels loaded with three different eccentricity values.

Numerical homogenization was also used in [4]. Since homogenization allows for a significant simplification of the computational models [1] and, at the same time, for a very accurate representation of complex plate cross-sections [1], the application of such techniques to the corrugated cardboard packaging becomes a very urgent task. As soon as the homogenized models begin to take into account the creases, cuts and other local effects of the plates, this technique begins to take on a very practical character. The authors used a very practical application of homogenization (already presented in work [1]) extended by also modeling cases containing all local effects resulting from production and processing. The presented approach can be successfully used to model the smear degradation in a finite element or to define the deterioration of stiffnesses on the crease or perforation line.

On the other hand, article [5] presented the important issue of thin facing wrinkling in sandwich panels with a soft core. The local loss of stability in thin facings obviously reduces the load-bearing capacity of the composite panels. Therefore, it is very important to correctly define under what conditions and for what loads this effect is activated in real structures. The paper compares the classic solutions to the problem of facing instability based on an eluted homogeneous and isotropic half-space (i.e., the soft core of the plate). The paper also discusses the use of an orthotropic core, in line with the classic solution of an isotropic core.

Corrugated board was analyzed again in [6]. The authors focused on the load-bearing capacity of corrugated cardboard packaging in a specific configuration of packaging flaps. The raised problem is particularly important in the corrugated board packaging industry, where more and more advanced numerical tools are used to design and estimate the load capacity of its products. Therefore, numerical analyses are becoming a common standard in this branch of production. Because the experimental results showed a significant reduction in the static load-bearing capacity of the package in the case of shifted flap creases, the study investigated the impact of the specific flap configuration on the strength of the box. An updated analytical and numerical approach was used to predict package strength with different flap offsets. The results obtained by the model presented in this paper were also verified with satisfactory compliance with the experimental data.

Paper [7] presented an issue that was partially discussed already in previous works in this series, namely plate edge crushing [2,3] and the use of optical extensometers [2] to measure displacements and deformations on the external surfaces of the tested samples. As is known in the plate edge crush tests, the biggest obstacle is obtaining a reliable measurement of displacements and deformations in the sample. Therefore, the use of video extensometry allowed the authors to develop a method that not only allows the reliable measurement of displacements, but also the identification of the full orthotropic stiffness matrix of the material. This was achieved through the innovative use of two samples: (a) traditional and cut across the wave direction of the corrugated core, and (b) cut at an angle of 45°. The obtained results were finally compared with the results obtained in the homogenization procedure [1,4] of the corrugated board cross-section.

Corrugated cardboard was also analyzed in two further studies [8,9]. In work [8], the authors focused their attention on the palletization of corrugated cardboard packaging, while in [9], on a rather unusual corrugated cardboard product, i.e., furniture. The first article examined the effect of the stiffness of the top deck of the pallet on the compressive strength of a corrugated board box as a function of the initial thickness of the top deck, the wood grade of the pallet, the size of the box and the grade of the cardboard. The second article focused on optimizing the stool structure by removing material zones in places where the fewest stresses occur. Interestingly, the work [9] also used homogenization methods similar to those presented in [1,4]. The presented results demonstrate the utility of homogenization techniques as an aid in the design process of whole structures made of corrugated cardboard.

A slightly different issue was presented in [10], where the authors focused on the construction of connections in a composite beam made of aluminum and wood. The load capacity, the type of failure and the load slip reaction of reinforced and unreinforced screw connections were examined. It has also been proven that the tested stiffness and strength of connections can be practically used for the correct design and numerical modeling of aluminum–wooden composite beams with reinforced bolted connections.

The topic related to the mechanics of paper and cardboard also appeared in [11], where the authors presented the effect of impregnation of the paper core with acetylated starch on the mechanical properties and energy absorbed in the three-point bending test of wood-based honeycomb panels, under changing temperature and relative air humidity conditions. The paper presented the results of extensive research on materials, various combinations of coatings, core cell geometry and different qualities of cardboard. The results of the experiment and their statistical analysis showed a significant relationship between the impregnation of paper with modified starch and its mechanical properties. In general, this observation obviously allows for the optimization of furniture boards and their further lightweighting.

Selected homogenization methods used for corrugated core materials presented in previous studies [1,4,9] have been systematically summarized in [12]. The homogenization methods presented in this work refer to materials with a lattice core, but their use for materials with a corrugated core is also possible. In both cases, structures made of plates containing structural cores are both light and very stiff. Without the use of homogenization, only conventional methodologies remain based on numerical approaches such as FEA (finite element analysis) and high-performance computational tools, including ANSYS and ABAQUS. However, they require a high computational power in each case of modeling complex core geometries. That is why it is so important to correctly apply the appropriate homogenization method to simplify the model and speed up the calculations, while maintaining the maximum fidelity of the simplified model in relation to the real model.

Last but not least, article [13] in our Special Issue presented the method of modeling the combustion of a popular material—aluminum. The authors conducted a study of aluminum powder in order to isolate the aluminum combustion process and determine an adequate representation of this process. The charges of various masses were investigated, determining the size of the cloud and previously unpublished results of the component ratio in the Al and air mixture. The obtained results of the numerical analysis as well as those obtained from the experimental tests were in good agreement.

To summarize, the problems related to the mechanics of corrugated and composite materials discussed in this Special Issue do not exhaust the topic but are only a small part of this broad topic. All the presented works follow the trend of modern scientific research on materials with a soft core (corrugated, lattice, etc.) and composites, as well as the practical use of homogenization techniques of structures made of these materials.
